# Integrated Stirred-Tank Bioreactor with Internal Adsorption for the Removal of Ammonium to Enhance the Cultivation Performance of *gdhA* Derivative *Pasteurella multocida* B:2

**DOI:** 10.3390/microorganisms8111654

**Published:** 2020-10-24

**Authors:** Siti Nur Hazwani Oslan, Joo Shun Tan, Sahar Abbasiliasi, Ahmad Ziad Sulaiman, Mohd Zamri Saad, Murni Halim, Arbakariya B. Ariff

**Affiliations:** 1 Bioprocessing and Biomanufacturing Research Centre, Faculty of Biotechnology and Biomolecular Sciences, Universiti Putra Malaysia, Serdang, Selangor 43400, Malaysia; hazwanioslan@gmail.com; 2 Faculty of Bioengineering and Technology, University Malaysia Kelantan, Jeli Campus, Jeli, Kelantan 17600, Malaysia; ziad@umk.edu.my; 3 Bioprocess Technology, School of Industrial Technology, Universiti Sains Malaysia, Penang 11800, Malaysia; jooshun@usm.my; 4 Halal Products Research Institute, Universiti Putra Malaysia, Serdang, Selangor 43400, Malaysia; s.abbasiliasi@gmail.com; 5 Research Centre for Ruminant Diseases, Faculty of Veterinary Medicine, Universiti Putra Malaysia, Serdang, Selangor 43400, Malaysia; mzamri@upm.edu.my; 6 Department of Bioprocess Technology, Faculty of Biotechnology and Biomolecular Sciences, Universiti Putra Malaysia, Serdang, Selangor 43400, Malaysia; murnihalim@upm.edu.my

**Keywords:** cation-exchange resin, adsorption, removal, ammonium, cell viability, mutant gdhA *P. multocida* B:2

## Abstract

Growth of mutant gdhA *Pasteurella multocida* B:2 was inhibited by the accumulation of a by-product, namely ammonium in the culture medium during fermentation. The removal of this by-product during the cultivation of mutant gdhA *P. multocida* B:2 in a 2 L stirred-tank bioreactor integrated with an internal column using cation-exchange adsorption resin for the improvement of cell viability was studied. Different types of bioreactor system (dispersed and internal) with resins were successfully used for ammonium removal at different agitation speeds. The cultivation in a bioreactor integrated with an internal column demonstrated a significant improvement in growth performance of mutant gdhA *P. multocida* B:2 (1.05 × 10^11^ cfu/mL), which was 1.6-fold and 8.4-fold as compared to cultivation with dispersed resin (7.2 × 10^10^ cfu/mL) and cultivation without resin (1.25 × 10^10^ cfu/mL), respectively. The accumulation of ammonium in culture medium without resin (801 mg/L) was 1.24-fold and 1.37-fold higher than culture with dispersed resin (642.50 mg/L) and culture in the bioreactor integrated with internal adsorption (586.50 mg/L), respectively. Results from this study demonstrated that cultivation in a bioreactor integrated with the internal adsorption column in order to remove ammonium could reduce the inhibitory effect of this by-product and improve the growth performance of mutant gdhA *P. multocida* B:2.

## 1. Introduction

*Pasteurella multocida* type B:2 known as Gram negative bacterium caused a hemorrhagic septicemia (HS) disease in cattle and buffaloes associated with morbidity leading to colossal losses to farmers and nation. An attenuated (mutant) *gdhA* derivative *P. multocida* B:2 has been developed by Sarah et al. [1] in order to control the HS disease. In addition, the mutant strain was stable and did not revert back to virulence (Hazwani et al., 2014) and established to maximize the survival rate, storage stability, and activity of the bacterial cells of live vaccine [2]. However, ammonium is accumulated, as a by-product, in the culture during the batch cultivation of mutant *P. multocida* B:2, which inhibits the growth and reduce the cell viability [2]. In pentose phosphate cycle, histidine metabolism is one of the amino acid biosynthesis and this metabolism might relate to the histidine utilization Hut pathways [3]. In this pathway, the bacterium is able to degrade histidine to ammonia. Furthermore, the Hut pathway is fundamentally a catabolic pathway that allows cells to use histidine as a source of carbon, energy, and nitrogen.

In environmental study, various methods have been developed to eliminate the ammonium ion in contaminated water. The methods include air stripping [4], ion exchange resins [5], adsorption [6], chemical precipitation [7], and nitrification reactions [8]. The use of cation-exchange resin for in situ removal of ammonium during batch cultivation of mutant *P. multocida* B:2 has been demonstrated to improve the viability of mutant *P. multocida* B:2 as discussed by Oslan et al. [2]. However, the use of dispersed resin in bioreactor for in situ adsorption of inhibitory metabolites may cause direct shear force from the impeller, which affects the stability and efficiency of the resins [9]. In addition, the bioreactor could carry out in situ adsorption of ammonium attached to the resin efficiency during fermentation and when the resin fluidized in the internal adsorbent provided better environmental conditions, such as pH level, temperature, aeration rate, and dissolved oxygen, as compared with external adsorbent could be achieved.

The stirred-tank bioreactor equipped with an internal adsorption column has been applied for improvement of the performance of fermentation subjected with by-product inhibition. In such a technique, the resin with the ability to adsorb the target by-product was entrapped in an internal adsorption column with no direct contact with the impeller in the bioreactor. The use of the internal column allows the fluidization of porous adsorbent resins, so in turn, the surface of adsorbent in the column can be increased. In addition, the use of an internal adsorption column allows the cells culture to flow freely through the column, while the product or by-product is captured at the same time [10].

In the previous study by Oslan et al. [11], they have shown the primary data on growth of mutant gdhA P. multocida B:2 were inhibited by the accumulation of by-product ammonium in the culture medium. The results from the study have demonstrated that ammonia accumulated in a culture of gdhA derivative *P. multocida* B:2 could be removed by the adsorption onto cation-exchange resins in shake flask. Hence, this study highlights the removal of by-product ammonium during the cultivation of mutant gdhA P. multocida B:2 in a 2 L stirred-tank bioreactor integrated with internal column using cation-exchange resin for improvement of cell viability mutant gdhA *P. multocida* B:2. The objective of this study was to develop an integrated stirred-tank bioreactor with an internal adsorption column for in situ removal of ammonium from the culture of mutant *P. multocida* B:2 for the enhancement of the growth performance in terms of cell count and viability. For comparison, the fermentations in a stirred-tank bioreactor without resin and with dispersed resin were also performed.

## 2. Materials and Methods

### 2.1. Microorganism and Inoculum Preparation

The *gdhA* derivative of *P. multocida* B:2 was cultured on blood agar base and incubated at 37 °C for 48 h. The single colony isolated from the petri dish was inoculated into flask containing 10 mL of yeast extract-dextrose with histidine (YDB-His) medium. This is the optimal medium for the production of mutant *P. multocida* B:2 [11]. The medium consisted of (g/L): yeast extract, 15.6; dextrose, 1.9; sodium chloride, 3; sodium dihydrogen phosphate, 2.5; and histidine, 3.1 supplemented with 60 mg/L streptomycin and 50 mg/L kanamycin. All chemicals were obtained from Sigma-Aldrich (St. Louis, MA, USA). A 5% of inoculum was inoculated into 100 mL YDB-His medium and incubated in an orbital shaker (Infors, GmbH, Germany) at 37 °C and agitated at 250 rpm for 16 h. The culture was standardized as an inoculum for the fermentation in bioreactor.

### 2.2. Stirred-Tank Bioreactor Design and Internal Column Adsorption System

**Figure 1:**
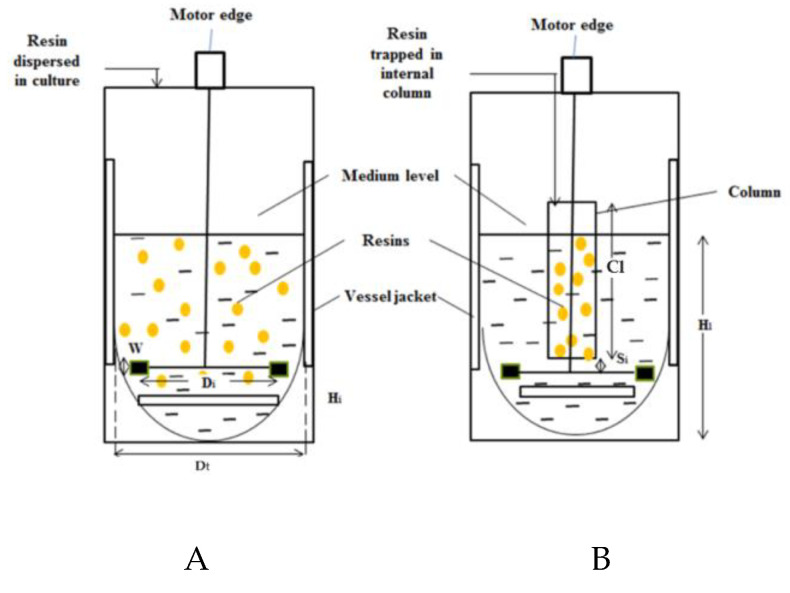
Schematic diagram of the stirred-tank bioreactor (**A**) without and (**B**) with internal column adsorption.

A 2 L stirred-tank bioreactor with a working volume of 1 L was used in this study. The bioreactor consisted of a double jacketed borosilicate glass vessel, four baffles, and a stainless-steel top plate with several opening ports for sampling and electrodes (BIOSTAT, B. Braun Biotech International, GmbH, Munich, Germany). Relative dimensions of the stirred-tank bioreactor used in this study, with and without internal adsorption column, are shown in Figure 1. The total length between the sparger and the motor edge was 13.6 cm. The glass vessel was concave at the bottom to eliminate dead-spots. The bioreactor was equipped with a single six-bladed Rushton turbine (RT) impeller located at 3.0 cm from the oval bottom of the vessel. The impeller was spaced 2.0 cm between the air sparger.

Table 1 shows the descriptions of the RT impeller and 2 L stirred-tank bioreactor. For experiments using internal column in bioreactor, the column was packed with the position of impeller below the column. The resins were then added into the column prior to autoclaving. The adsorption study was conducted simultaneously in batch fermentation from the culture throughout the whole fermentation process. The impeller was spaced 1.5 cm between the columns. The stirred-tank bioreactor was equipped with antifoam level, temperature, pH, and dissolved oxygen tension (DOT) control systems. The temperature of the culture was maintained at 37 °C, and the temperature was continuously measured by a platinum resistance thermometer with standard Pt100 probe (PT100, Mettler Toledo, Greifensee, Switzerland). The DOT in the culture was measured by a polarographic oxygen probe (InPro 6900, Mettler Toledo, Switzerland). After sterilization at 121 °C, 15 psi for 15 min, the probe was calibrated by sparging with nitrogen gas to set the DOT at 0% saturation, and then, the DOT was set to 100% saturation by sparging with filtered air for several hours until oxygen was saturated. The pH of the culture was monitored continuously by a pH (Ag/AgCl_2_) electrode (InPro3253/225/PT100 Mettler Toledo, Switzerland), but the pH was not controlled during the fermentation. The dissolve oxygen tension (DOT) was automatically adjusted to the set level and the impeller speed was fixed according to the design of experiment. The different agitation was studied at 200 rpm, 300 rpm, 400 rpm, and 500 rpm for bioreactor without resin and dispersed resin, followed by 700 rpm for cation-exchange resin in column. All experiments were performed in triplicate, each being repeated at least three times.

### 2.3. Cultivation Experiments

The Amberlite 86 cation-exchange resins (Sigma-Aldrich, St. Louis, MA, USA) were selected for this study based on the highest ammonium adsorption capacity from a previous study by Oslan et al. [11]. The Amberlite 86 cation-exchange resins at 10 g/L were prepared and washed with deionized water to remove the impurities. The washed resins were directly inserted into the column positioned in the bioreactor before being autoclaved at 121 °C for 15 min. The experiment was carried out after the cooling down of the bioreactor; the inoculum was transferred into the bioreactor with the supplementation of 60 mg/L streptomycin and 50 mg/L kanamycin. Kanamycin was added to prevent the release of kanamycin cassette by the cells, whereas streptomycin was added to avoid the growth of foreign cells in the culture.

### 2.4. Determination of Volumetric Oxygen Transfer Rate (k_L_a)

The dynamic gassing out method in non-fermentative system was used in this study for the determination of k_L_a [12]. Initially, the dissolved oxygen in test liquid medium, without microorganism, was purged with nitrogen gas. Once dissolved oxygen tension (DOT) readout stabilized at 0%, compressed air was sparged in, and the gradual rise of DOT was monitored and recorded at 5 s interval until equilibrium saturation achieved. The k_L_a was determined in a stirred-tank reactor with and without internal adsorption column with the presence and absence of resins. The following oxygen mass transfer model was used for the determination of k_L_a:

dC_L_/dt *=* K_L_a(C_E_ − C_L_) (1)

where C_L_ is the dissolved oxygen concentration and C_E_ is the saturated dissolved oxygen concentration in the solution. k_L_a was estimated from the slope of the straight line obtained from a plot of ln (C_E_ − C_L_) against the time.

### 2.5. Analytical Methods

The bacterial cell viability was expressed via serial dilution plate count technique and determined the dry cell weight (DCW). To determine the DCW of the culture sample, 10 mL of the sample culture from the bioreactor was centrifuged at 10,000× *g* at 4 °C for 15 min (Eppendorf, Hamburg, Germany), and the supernatant was used for glucose determination. The pellet of the cell was dried in an oven at 70 °C until a constant weight was obtained. The glucose concentration was analyzed using biochemistry analyzer (YSI 2700; Yellow Spring Instruments, OH, USA). The concentration of ammonium in the fermentation culture was determined using a Nessler method [13].

The cation-exchange resins and samples of cell culture were collected after the fermentation and examined under scanning electron microscope (SEM) (LEO 1455 VPSEM, Kensington, UK). For electron microscopic study, the cell culture was centrifuged at 10,000× *g* at 4 °C for 15 min (Eppendorf, Hamburg, Germany), and the pellet cells were fixed in 4% (*v*/*v*) glutaraldehyde for 4 h at 4 °C. Then, the fixed cells were washed three times for 10 min using 0.1 M sodium cacodylate buffer. After post-fixation in 1% (*w*/*v*) osmium tetroxide for 2 h at 4 °C, the cells were washed again and dehydrated with a series of increasing concentrations of acetone 35%, 45%, 55%, 75%, and 95% for 10 min; followed by 100% for 15 min for 3 times followed by sectioning using a Leica-Reichert Ultracut Ultramicrotome (Leica, GmbH, Wetzlar, Germany) and staining with uranyl acetate and lead citrate for 10 min. Then, the samples were mounting on stuck before viewing under SEM.

## 3. Results

### 3.1. Effect of Agitation Speed on Volumetric Oxygen Transfer Rate (k_L_a)

**Figure 2:**
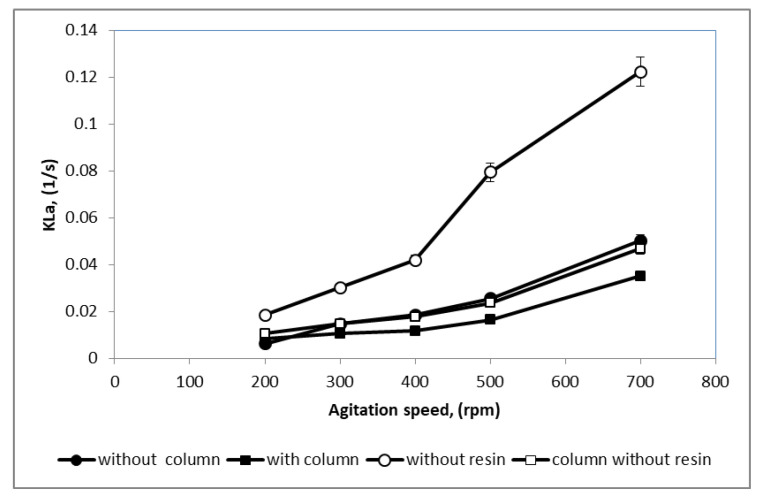
Comparable values of k_L_a for the 2 L stirred-tank bioreactor. (**○**) bioreactor without column no resin; (●) bioreactor with dispersed Amberlite IRC 86 resin (no column); (□) bioreactor with internal column without resin; (■) bioreactor with Amberlite IRC 86 resin in an internal column.

Impeller speed is an important factor that effects gas liquid mass transfer in an agitated bioreactor. Figure 2 represents experimentally obtained correlations for evaluation of k_L_a values for a 2 L stirred-tank bioreactor set up (i) without column and no resin, (ii) with dispersed resin (no column), (iii) with internal column without resin, and (iv) with resin in an internal column at different agitation speeds from 200 to 700 (rpm) with air flow rate fixed at 1 L/min in the 2 L stirred-tank bioreactor. The k_L_a values were found to increase with increasing impeller speed. The increasing values of k_L_a was due to the breakage of rising bubbles by the impeller blade and gave rise to more interfacial area for gas transfer, subsequently increasing oxygen transfer in the culture. For both bioreactor systems, with and without an internal adsorption column, k_L_a was significantly reduced with the presence of resins. At the same agitation speed, k_L_a for the bioreactor with an internal adsorption column was significantly lower as compared to the bioreactor without an internal column. In addition, the k_La_ values for the bioreactor with an internal adsorption column was not significantly different at all agitation speed tested. With the integration of the column and resins into the bioreactor, the mixing of gas bubbles in the broth was slowed down, and subsequently reduced the oxygen transfer to the cells.

### 3.2. Effect of Amberlite IRC 86 Resins on the Cultivation Performance of Mutant P. multocida B:2

**Figure 3:**
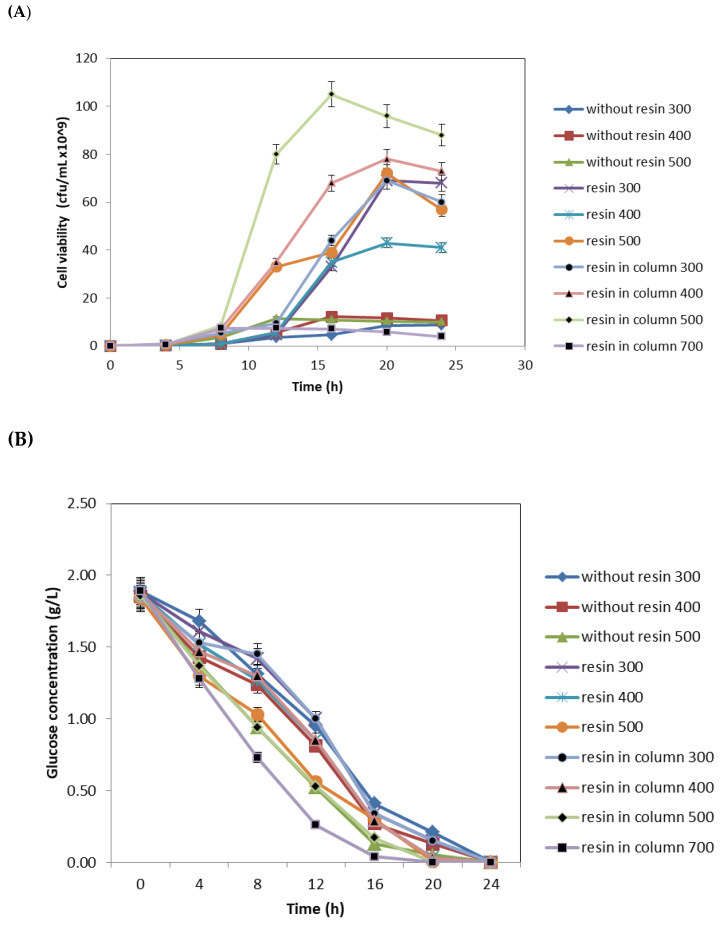

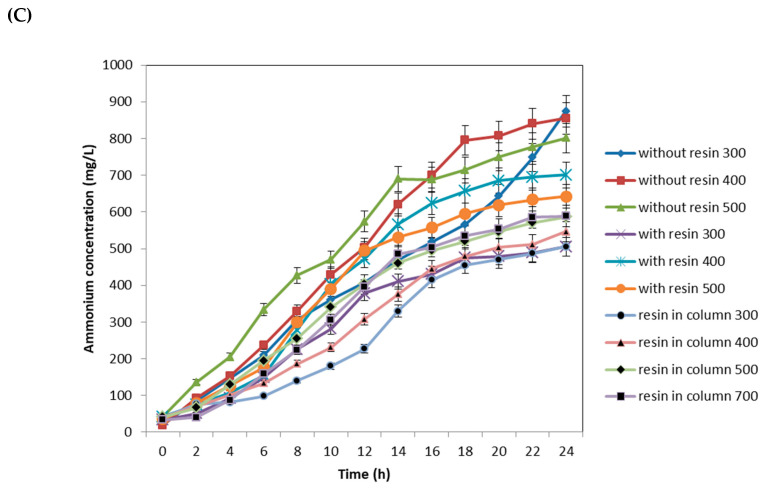
The time course of batch cultivation of *gdhA* derivative *Pasteurella multocida* B:2 with cell viability (**A**), glucose consumption (**B**), and ammonium concentration (**C**) in a 2 L stirred-tank reactor without the addition of resin, with dispersed Amberlite IRC 86 resin and Amberlite IRC 86 resin in internal column at different agitation speed.

Figure 3 shows the time course of batch cultivation of mutant *P. multocida* B:2 in a 2 L stirred-tank bioreactor (i) without the addition of resin, (ii) with dispersed resin, and (iii) with resin in an internal adsorption column at different agitation speed (300, 400, 500, and 700 rpm). Based on the growth performance in Figure 3A, cell viability in the culture without Amberlite IRC 86 resins significantly decreased in cell viability as compared with dispersed resins and resins in internal adsorption column. The maximum cell viability (1.05 × 10^11^ cfu/mL) in a stirred-tank reactor with internal adsorption column, agitated at 500 rpm, was obtained at 16 h. To further study the effect of agitation speed on the cell viability in a stirred-tank reactor with internal adsorption column, the agitation speed was increased to 700 rpm. The cell viability was significantly decreased at higher agitation speed (700 rpm). The cell viability was significantly increased after 8 h in all fermentations tested.

Table 2 shows the comparison of the cultivation performance of mutant *P. multocida* B:2 in three different types of stirred-tank reactor with a different set up. The highest cell viability in the system without internal adsorption column was 7.2 × 10^10^ cfu/mL, while the culture with resins in column showed much higher viable cells (1.05 × 10^11^ cfu/mL) in the system at the agitation speed of 500 rpm. The result also indicated that the cell viability was significantly different (*p* < 0.05) when resins were used in this study. In terms of glucose consumption, Figure 3B shows the glucose was completely consumed at 20 h and the biomass production increased markedly with time at 16 h for all fermentations tested (Figure 3B). In addition, the fermentation in an integrated system of stirred-tank bioreactor and internal column shows that the growth cycle with maximum cell viability production was archived at 20 h and 16 h fermentation, respectively.

Figure 3C shows the ammonium accumulated in the culture during the cultivation of mutant *P. multocida* B:2 in a 2 L stirred-tank reactor with different set up. The ammonium concentration in the bioreactor without resin was significantly increased with time, since a maximum concentration of 874.33 mg/L was achieved after 24 h at 300 rpm in which the culture pH was increased to pH 8.9. In the cultivation with resins in internal adsorption column, ammonium accumulation in culture medium was significantly reduced in which the highest concentration of ammonium was only 505.17 mg/L. In addition, the accumulation of ammonium at 16 h was 699.83 mg/L for reactor without resins, 557.67 mg/L in reactor with dispersed resin, and 494.5 mg/L for reactor with internal column. Hence, the cell viability increased with the decreased ammonium accumulation in the culture. This indicated that the addition of cation-exchange resins could reduce the ammonium accumulation in the culture medium by removal of the ammonium to a non-lethal level, as shown in Figure 3C. Hence, high concentration of cell viability of mutant *P. multocida* B:2 was achieved (1.05 × 10^11^ cfu/mL).

### 3.3. Effect of Cation-Exchange Resin on Cell Morphology of Mutant P. multocida B:2

**Figure 4:**
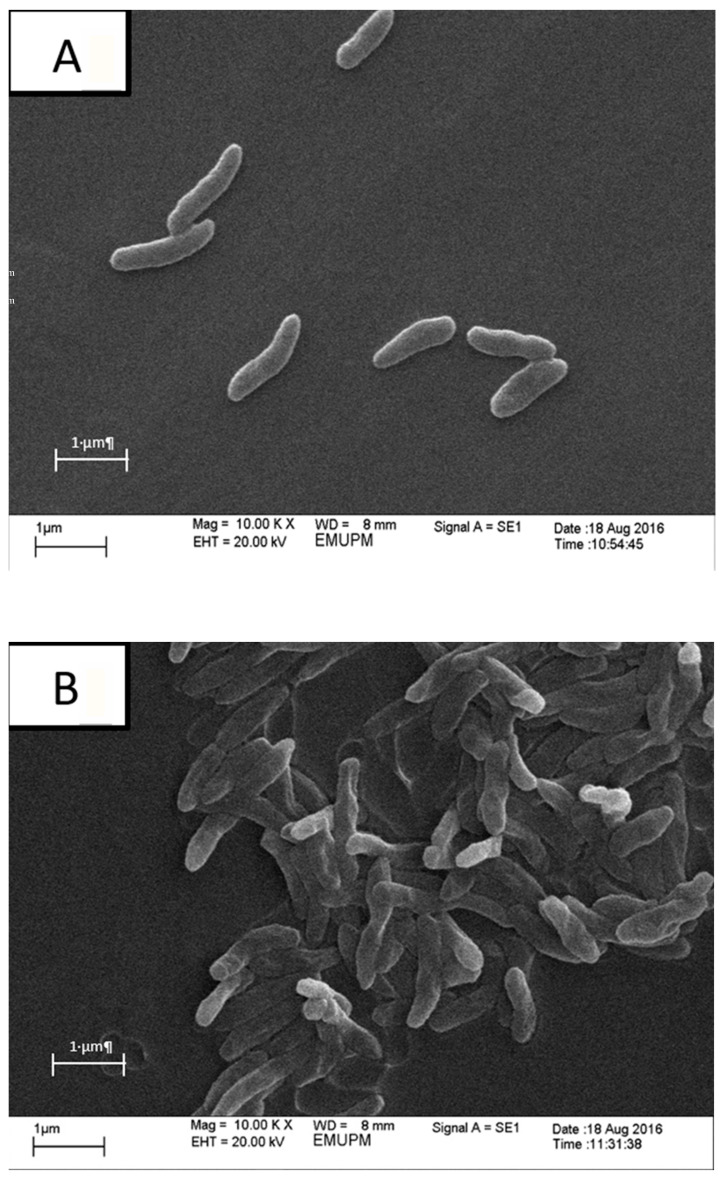

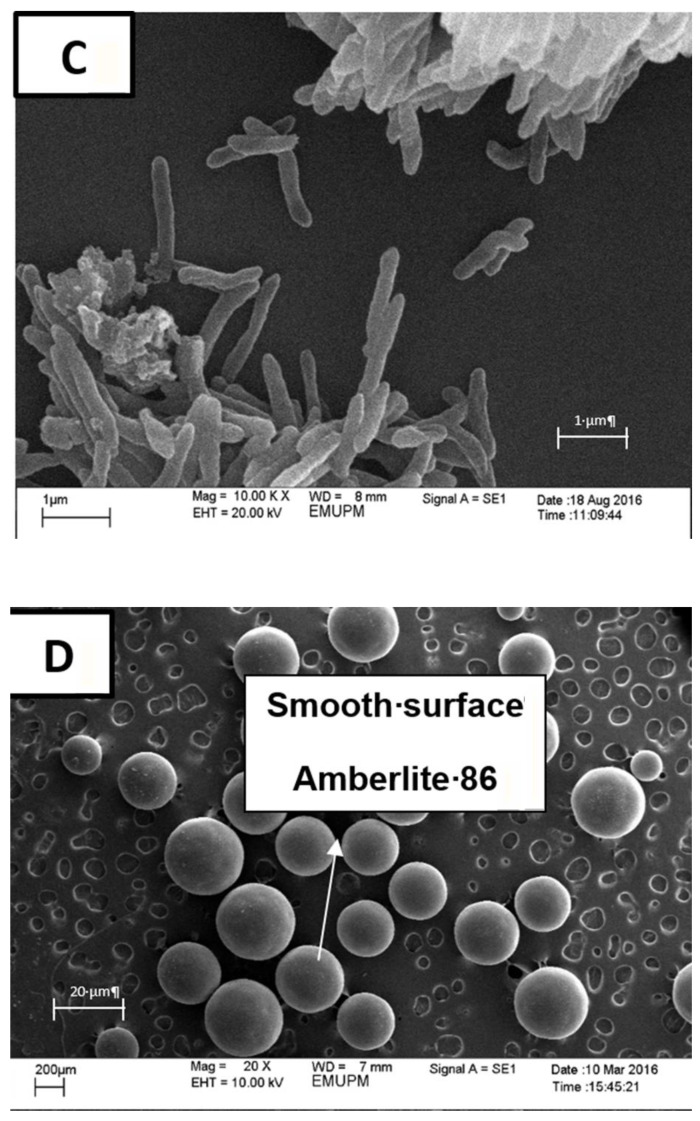

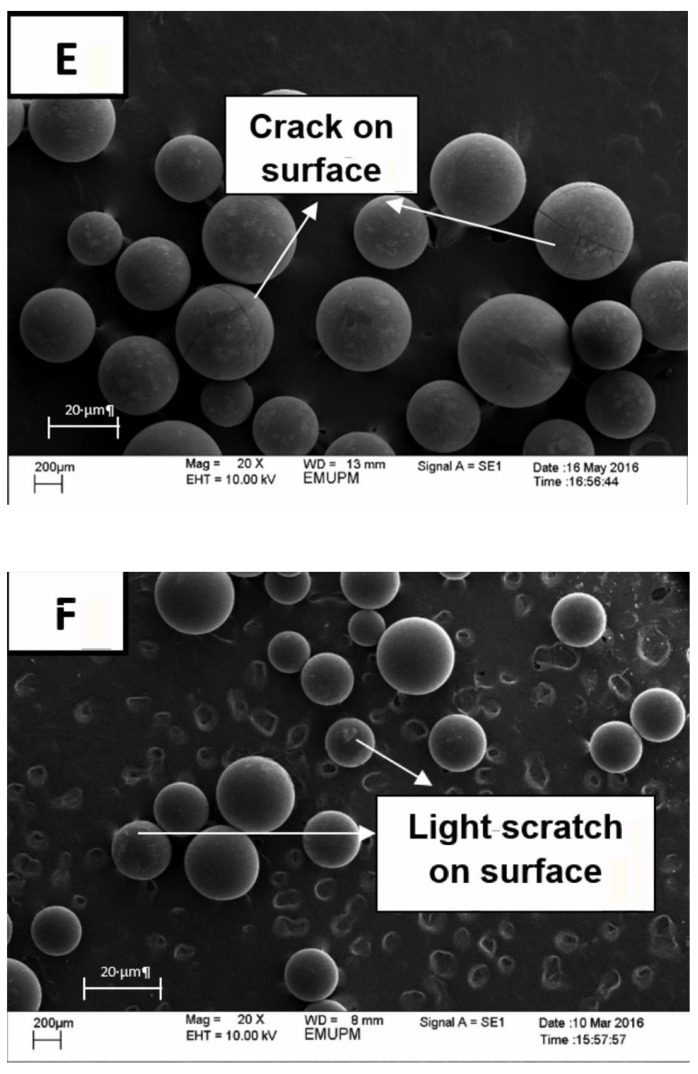
Scanning electron photograph of cell morphology mutant *gdhA P. multocida* B:2 (magnification ×10,000) in a 2 L stirred-tank bioreactor (**A**), without resin (**B**), with dispersed resin (**C**), with resin in internal column and surface structure resins Amberlite 86 (magnification ×200). (**D**) Control surface with smooth surface on resins. (**E**) Crack on the surface dispersed resins. (**F**) Light scratch on surface resins in internal column.

At the end of the cultivation of mutant *P. multocida* B:2 with the in situ addition of cation-exchange resin, the cells were examined under the scanning electron microscopy (SEM). Figure 4 shows SEM photograph (magnification ×10,000) of cell morphology mutant *P. multocida* B:2 that were cultivated in a 2 L stirred-tank bioreactor without resin, (control) (A), (B) stirred-tank bioreactor with dispersed resin, and (C) stirred-tank bioreactor with resin in internal column. The morphology of mutant *P. multocida* B:2 cultivated with resin and control was similar, which was long rod. SEM of *P. multocida* showed that they exist as colonies of rod-shape bacteria in cells cultivated in bioreactor without resin with an average cell length of 1 µm and cell width of 0.4 µm. Overall, the morphology of the cells that cultivated in the bioreactor without resin was apparently normal (Figure 4A), but the cells deformed (Figure 4B) when cultivated in bioreactor with dispersed resin and elongated (Figure 4C) were observed for cells when cultivated in the bioreactor with resin packed in the internal column.

### 3.4. Effect of Agitation Speed on Surface Of Cation-Exchange Resin

Figure 4 shows scanning electron photograph (magnification ×200) on surface of cation exchange resin Amberlite IR86 (A), control resin (B), dispersed resin (C), in internal column at 500 rpm. Cracked surface was observed for resin used in cultivation with dispersed resin (Figure 4E). Meanwhile, only a very light scratch was detected on the surface of resins that were packed in an internal adsorption column (Figure 4F). Resins packed in an internal column were protected from experiencing direct shear force from the impeller that may damage the surface and consequently may enhance ion-exchange capacity and provide greater available surface area for adsorption site. This observation supports the assumption made in Section 2 on the lower cell viability (7.2 × 10^10^ cfu/mL) of culture in dispersed resin systems compared to culture with resins packed in a column (1.05 × 10^11^ cfu/mL) that may be due to the influence of direct shear force. The result is in agreement to the study of Tan et al. [9] in which the bioreactor with a column had better performance in terms of growth and productivity.

## 4. Discussion

Results from this study have demonstrated that the use of a stirred-tank bioreactor with internal adsorption column packed with resins has successfully improved the cultivation performance of mutant *gdhA P. multocida* B:2, by removing the ammonium accumulated in the culture. In this study, the experiment was shown the effect of agitation speed on volumetric oxygen transfer rate (k_L_a) with the integration of column and resins into the bioreactor, it slowed down the mixing of gas bubbles in the broth, subsequently reducing the oxygen transfer to the cells. Faccin et al. [14] reported that low oxygen supply may reduce the tricarboxylic acid (TCA) cycle activity, due to both thermodynamic reasons and metabolic control by reducing power NADPH, and allow that part of the acetyl-CoA available. The oxidation via TCA cycle predominates under balanced growth conditions, with NADH being generated and used in biosynthesis or energy generation. When biosynthesis decreases due to lack of a nutrient, the TCA cycle activity also decreases due to the high NADH concentration, resulting in a decrease in acetyl- CoA oxidation via TCA cycle. Under the limitation of oxygen, the reducing power NADPH, the by-product of ammonium ion could be reduced. In addition, the cultivation performance of aerobic microorganism will also be affected in oxygen-limited culture [15]. In order to provide sufficient oxygen transfer from the gas to the liquid phase, a suitable degree of agitation that exerted no or less damage to the microbial cell must be selected.

Despite the lower k_L_a value observed with the addition of resin and column compared to the bioreactor system without the presence of resin and column (Figure 2), in this study, the agitation speeds were sensitive in shear rate level, which may cause damage to the bacterial cell. Although oxygen is essential to growth and production, at elevated concentrations it could also be toxic for a variety of microbes [16]. Most aerobic microorganisms have developed protective responses to tolerate environmental oxygen concentrations. In *E. coli* cultures, when oxygen concentration surpasses the air saturation level, the oxygen species (ROS) such as accumulated byproducts of aerobic metabolism reactivated [17]. In addition, the use of oxygen-enriched air has become an accepted practice to support the aerobic growth of cells in bioreactors, which increases the cell density and improves process productivity [16]. However, exposing cells to high oxygen concentrations is known to enhance the accumulation of byproduct and reach to harmful levels that can overwhelm and cause stress to the cells [18]. In order to provide sufficient oxygen transfer from the gas to the liquid phase, the optimum agitation speed that did not cause damage to the microbial cells must be selected. The results shows constant k_L_a ensures equal oxygen transfer rates at the various scales of operation.

The in situ removal of ammonium accumulation in bioreactor system with selected resins provided certain conditions that influenced the production of viable cell of mutant *gdhA P. multocida* B:2. The integrated bioreactor system in this study enhance growth of mutant *gdhA P. multocida* B:2 with high cell viability through the removal of ammonium by resin adsorption. In this system, the liquid enters the internal column through lateral perforations (flow through perforated column). It was shown that the continuous adsorption between resin and medium occurs during fermentation. This concept has been applied by Jahangiri-Rad et al. [19], where the continuous fixed bed study was carried out by using PAN-oxime-nano Fe_2_O_3_ as a sorbent for the removal of nitrate from aqueous solution. The other available adsorption methods using a column are packed-bed chromatography [20] and fluidized beds in a bioreactor [18]. However, in packed-bed mode, a long sample application time and initial removal of particulate material are required to prevent clogging of the bed. The integrated packed bed column in the bioreactor was also successfully applied by Yanagi et al. [21] to enable high density culture of hepatocytes for use as a hybrid artificial liver support system or a bioreactor system. In their system, a packed-bed reactor was packed with using collagen-coated reticulated polyvinyl formal (PVF) resin, and this system was applied to a primary cultivation of hepatocytes. In the aqueous solution, ammonia exists in two different forms, which are ammonium ion and ammonia molecule. Some of the ammonia will be adsorbed by the resins, but some will be released by the system to environment following to the oxygen stream. Hence, quantification of the ammonia adsorbed by the resin is recommended for future study.

In this study, the effect of macroporous Amberlite 86 resin on the morphology of mutant *P. multocida* B:2 was investigated under SEM. The morphology of cell culture mutant was not changed throughout the cultivation. Moreover, the adsorption capacity of ammonium by the resins was dependent on the size of resins [22]. This indicates that without the direct shear force from the impeller, the resin has a higher ion-exchange capacity and greater available surface area. On the other hand, larger and higher density particles move to the bottom of the column in a bioreactor, forming a stable and uniform expansion. In addition, the use of small diameter resins and enlarged pores could be reduced the transport resistance, resulting in an increase in the dynamic adsorption capacity at high flow velocities in culture [9]. Higher density and larger size particles increase the operation flow rate, resulting in a reduction in the processing time. In this study, the Amberlite granules were not covered by *P. multocida* cells, as the cell surface charge of *P. multocida* cells was a negative charge [23], which would be repelled by Amberlite cation-exchange resin.

During the in situ adsorption in a bioreactor, the direct shear force from the impeller may affect the resin surface. Dispersed resin in a bioreactor will be damaged due to the direct contact with impeller in a bioreactor as well as higher collision between one another at increasing impeller speed. In contrast, direct shear effect can be avoided with the application of an internal column as the surface of resin can be fully covered by the column, and the adsorption may continuously take place in the bioreactor without direct contact between resins and the impeller. In addition, the influence of direct shear force on the cells resulted from the collision with resins, as in the bioreactor with the dispersed resins, could be avoided to yield higher viable cells in the integrated column system. The adsorption capacity in the culture could be increased due to the increment of the resin surface area in the trapped column.

## 5. Conclusions

Overall, this study has demonstrated that the concept of a stirred-tank bioreactor with an internal adsorption column packed with resins have successfully improved the cultivation performance of mutant gdhA *P. multocida* B:2 by continuously removing ammonium accumulated in the culture. The in situ removal of ammonium accumulation in a bioreactor system with selected resins influenced the production of viable cell of mutant gdhA *P. multocida* B:2. The accumulation of ammonium in the culture was significantly reduced with the addition of resin with the ability to adsorb ammonium during the cultivation process in the internal column with absorbent resin. The highest concentrations of ammonium accumulated in cultivation media without resin, with dispersed resin (without internal adsorption column), and in cultivation with internal adsorption column packed with resin were 801 mg/L, 642.50 mg/L, and 586.50 mg/L, respectively. The improvement in cell viability was significantly different at *p* < 0.05 as compared with cultivation with dispersed resin (without internal adsorption column) and without resin, where the highest viable cell numbers were 1.05 × 10^11^ cfu/mL, 7.2 × 10^10^ cfu/mL, and 1.25 × 10^10^ cfu/mL, respectively. To date, this kind of study has never been reported for any live bacterial vaccine aiming for the improvement of cell growth.

## Figures and Tables

**Table 1:** Descriptions of Rushton turbine (RT) impeller and 2 L stirred-tank bioreactor.

Descriptions	Value
D_i,_ impeller diameter(cm)	5.5
D_t_, tank diameter (cm)	13.0
Hi, height impeller (cm)	5.6
Hl, height liquid (cm)	10.0
Li, impeller blade length (cm)	1.4
W_i_, impeller width (cm)	1.0
N, number of blades	6
C_l_, Column length (cm)	15

D_i_ impeller diameter; D_t_, tank diameter; Hi, height impeller; Hl, height liquid; Li, impeller blade length; W_i_, impeller width; C_l_, Column length.

**Table 2:** Effect of culture *gdhA* derivative *P. multocida* B:2 with column and with resin in a 2 L stirred-tank bioreactor.

	Kinetic Parameters			
Media: YDB-His Agitation Speed: 500 rpm	Max Viable Cell (cfu/mL)	X_max_ (mg/mL)	Specific Growth Rate, *µ* (h^−1^)	Productivity, *Pr* (mg/mL.h)
without column and without resin	1.23 × 10^10 a^	3.89 ± 0.05	0.505 ± 0.0882	0.243
without column and with resin	7.2 × 10^10 b^	3.97 ± 0.08	0.554 ± 0.0925	0.1985
with column and with resin	1.05 × 10^11 c^	4.04 ± 0.04	0.584 ± 0.095	0.2525

Values are mean ± SD (*n* = 3), ^a–c^ superscript with different letter is significantly different at *p* < 0.05.
